# Cognitive Neuroscience in Space

**DOI:** 10.3390/life4030281

**Published:** 2014-07-03

**Authors:** Gabriel G. De la Torre

**Affiliations:** Department of Psychology, University of Cadiz, Campus Río San Pedro S/N, 11510 Puerto Real (Cadiz), Spain; E-Mail: gabriel.delatorre@uca.es; Tel.: +34-646-287398

**Keywords:** cognitive neuroscience, space psychology, human space flight, human factors

## Abstract

Humans are the most adaptable species on this planet, able to live in vastly different environments on Earth. Space represents the ultimate frontier and a true challenge to human adaptive capabilities. As a group, astronauts and cosmonauts are selected for their ability to work in the highly perilous environment of space, giving their best. Terrestrial research has shown that human cognitive and perceptual motor performances deteriorate under stress. We would expect to observe these effects in space, which currently represents an exceptionally stressful environment for humans. Understanding the neurocognitive and neuropsychological parameters influencing space flight is of high relevance to neuroscientists, as well as psychologists. Many of the environmental characteristics specific to space missions, some of which are also present in space flight simulations, may affect neurocognitive performance. Previous work in space has shown that various psychomotor functions degrade during space flight, including central postural functions, the speed and accuracy of aimed movements, internal timekeeping, attentional processes, sensing of limb position and the central management of concurrent tasks. Other factors that might affect neurocognitive performance in space are illness, injury, toxic exposure, decompression accidents, medication side effects and excessive exposure to radiation. Different tools have been developed to assess and counteract these deficits and problems, including computerized tests and physical exercise devices. It is yet unknown how the brain will adapt to long-term space travel to the asteroids, Mars and beyond. This work represents a comprehensive review of the current knowledge and future challenges of cognitive neuroscience in space from simulations and analog missions to low Earth orbit and beyond.

## 1. Introduction

The human brain evolved very rapidly after our ancestors began to stand up and walk. This standing position favored the use of hands and tools, a key aspect in the ongoing human evolutionary process. Our brain has the ability to continuously adapt to new situations and demands from our environment. Like children progressing through their developmental stages, astronauts have to adapt to a completely new and weightless environment. Balance, movement and other brain functions are affected under zero gravity or microgravity conditions. Space cognitive neuroscience seeks to understand how the brain and mind react to the special environmental conditions of space. Among the factors inherent to the space environment that may affect the human brain and mind are microgravity, radiation, weightlessness, acceleration, noise and stress. The study of the human brain in space has become an important subject in recent years. The first studies on space neuroscience go back to 1962 during the Russian Vostok-3 mission, when some sensory-motor studies were carried out. On Earth, new brain imaging techniques, neuropsychological assessment tools and other physiological measures have been developed to enable very detailed studies of brain activity and cognitive functioning. For neuroscientists, as well as psychologists, it is of high relevance to understand the underlying neurocognitive and neuropsychological parameters of space flight. Unfortunately, standard brain imaging techniques (e.g., functional magnetic resonance imaging (fMRI)) are not applicable in space, due to the payload restrictions of space missions and costs. The European module “Columbus”, a part of the International Space Station (ISS), was equipped with an electroencephalography (EEG) system as a tool to research the link between weightlessness and central nervous system (CNS) activity [1]. Neurocognitive tests, electrophysiological measurements and other related methods are commonly used in space to assess brain activity, neurocognitive and behavioral status and the mental health of astronauts.

Earth-based research may also help to further our understanding of how the brain functions in space. This is achieved using so-called space analog environments and space simulations; both can be defined by their extreme environmental characteristics. The most well-known space analogs are the Antarctic and Arctic stations and desert and submarine installations. At these facilities, crewmembers spend months in isolation and harsh weather conditions performing a variety of tasks and procedures similar to those carried out in space missions. Other research paradigms that help in the field of space neuroscience include bed-rest experiments. When astronauts return from a long flight, their bodies need days to recuperate from the effects of living in weightlessness. In bed-rest experiments, the subjects remain in supine position on a bed for long periods, with the head titled slightly downwards while performing different tasks. Bed-rest studies resemble some aspects of weightlessness, allowing scientists to probe how the body reacts to microgravity and to test methods for keeping future astronauts fit and healthy.

**Figure 1:**
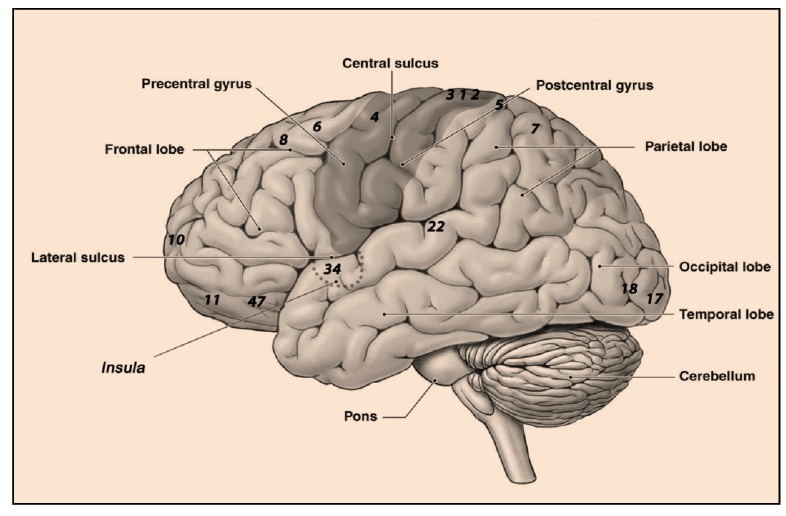
Cortical areas of the human brain affected in space. Numbers used for anatomical reference correspond to Brodmann areas. 1, 2 and 3: primary somatosensory cortex; 4: primary motor cortex; 5: somatosensory association area; 6: premotor cortex; 7: parietal cortex; 8: frontal eye field; 10, 11: prefrontal cortex; 17, 18: visual cortex; 22: auditory cortex; 34: dorsal entorhinal cortex.

A well-known threat to the success of space missions is the inadequate and ineffective performance of the crew. Results from Earth-based research highlight the importance of studying the effects of stress on cognitive performance. Cognitive and perceptual motor performances deteriorate under stress [2,3,4]. We can thus expect similar effects in the stressful environment of a space mission and in extreme environments and simulations. Previous work has shown that various psychomotor functions are degraded during space flight, among them central postural functions [5,6] involving hierarchically organized brain areas, including motor cortex in frontal lobes, basal ganglia, vestibular system in the midbrain and cerebellum, the speed [7,8,9] and accuracy of aimed movements [5,10] associated, among others, with primary motor cortex, cerebellum and visual cortex, internal timekeeping [11] related to prefrontal cortex and striatum, attentional processes [12] distributed in different brain areas, such as frontal and parietal cortex, superior colliculi subcortical region, frontal eye field and anterior cingulate cortex, limb position sense [13,14], including the primary somatosensory cortex and cerebellum, and the central management of concurrent tasks [15] involving mainly prefrontal, temporal and parietal cortex and basal ganglia (Figure 1). Such psychomotor deficits have been implicated as the causes of accidents in space [16]. It remains unknown to what extent these observed deficits might change during long-term space missions, as on a flight to Mars, for example. Results from Mars-500, one of the longest space mission simulations ever, revealed that a variety of neurological and psychological factors, such as circadian rhythms and social behavior, were affected by characteristics of the mission and the special environment [17].

## 2. Neuroscience in Space

### 2.1. Microgravity and Space Motion Sickness

**Figure 2:**
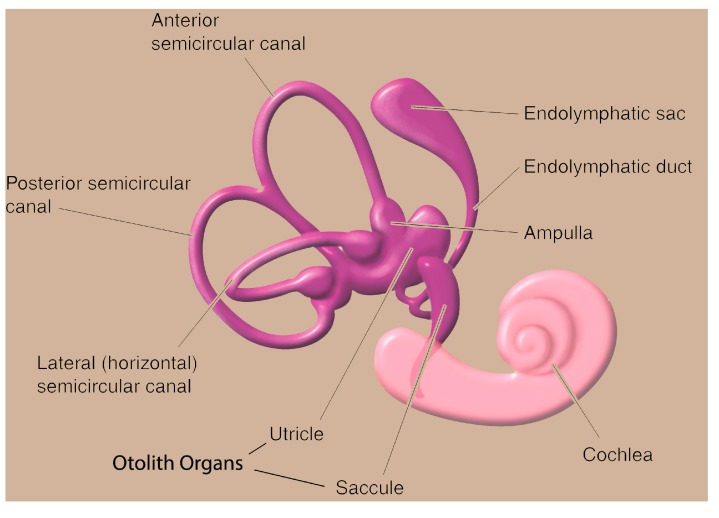
The vestibular system.

Gravity has shaped life on Earth. It is perceived by all organisms, from unicellular forms to humans, determines our orientation in space and helps control posture. We have specialized organs, such as the vestibular system in the inner ear, for gravity perception. Sensory information about motion, equilibrium and spatial orientation is provided by the vestibular apparatus in each ear, which includes the utricle, saccule and three semicircular canals. The utricle and saccule detect gravity (vertical orientation) and linear movement. The semicircular canals, which detect rotational movement, are located at right angles to each other and are filled with a fluid called endolymph. When the head rotates, the direction is sensed by a particular canal. The endolymphatic fluid within the canal lags behind, due to inertia, and exerts pressure against the canal’s sensory receptors. The receptors then send impulses to the brain conveying information about movement. When the vestibular organs on both sides of the head are functioning properly, they send symmetrical impulses to the brain (Figure 2).

Balance information provided by the peripheral sensory organs—eyes, muscles and joints and the two sides of the vestibular system—is relayed to the brain stem. There, it is processed and integrated with learned information contributed by the cerebellum (the coordination center of the brain) and the cerebral cortex (the thinking and memory center). A person can become disoriented if the sensory input received from his or her eyes, muscles and joints, or vestibular organs conflict with one another, and this can produce what is called motion sickness (Table 1). Approximately 70% of astronauts experience space motion sickness (SMS) during the first week of the mission [18].

**Table 1:** NASA classification of space motion sickness (SMS) according to the severity of symptoms.

None	No signs or symptoms reported
**Mild**	One or more transient symptoms No operational impact All symptoms resolved in 36–48 h
**Moderate**	Several symptoms of a persistent nature Minimal operational impact All symptoms resolved in 72 h
**Severe**	Several symptoms of a persistent nature Significant performance decrement Symptoms may persist beyond 72 h

On Earth, gravity is also a neural reference that influences how we perceive an object’s movement and orientation, an ability frequently disrupted in space. For example, moving the head while looking at a control panel can induce the perception that instruments are being displaced [19]. Perception is a cognitive process, and the way we perceive objects in the environment affects our perception of that environment. In space, this is a challenge, due to microgravity. Microgravity alters how we perceive the environment, producing illusory perceptions that have persistent after-effects in astronauts who spend long periods in space. If perception is affected by microgravity conditions, gravity may actually have an inner representation in the brain that is needed for important functions, such as proper motor control and motor planning. This has been confirmed by studying the effects of microgravity on covert and overt actions. Furthermore, this inner representation may affect anticipation actions [18]. However, microgravity does not affect verticality perception. Indeed, systematic behavioral observations of the motor behavior of astronauts during short-duration space flight suggest that they preferably align their posture with the vertical polarity of the spacecraft [19,20].

### 2.2. Space, Brain Activity and Sleep

#### 2.2.1. Brain Activity and Sleep in Space

Human sleep occurs with circadian (*circa* = about, and *dis* = day) periodicity. Circadian clocks evolved to maintain appropriate periods of sleep and wakefulness, in spite of the variable amount of daylight and darkness in different seasons and at different places on the planet. To synchronize physiological processes with the day-night cycle (photoentrainment), the biological clock must detect decreases in light levels as night approaches.

The receptors that sense these light changes include poorly understood cells within the ganglion and amacrine cell layers of the retina that project to the suprachiasmatic nucleus of the hypothalamus. Other structures are also implicated, such as the pineal gland, which synthesizes the sleep promoting neurohormone, melatonin (N-acetyl-5-methoxytryptamine), from tryptophan and secretes it into the bloodstream to help modulate the brainstem circuits that ultimately govern the sleep-wake cycle. Melatonin synthesis increases as light intensity decreases through the night. To study brain activity, especially during sleep, neuroscientists use EEG. The EEG detects abnormalities in the waves and electrical activity of the brain. During the procedure, electrodes consisting of small metal discs with thin wires are pasted on the scalp. The electrodes detect tiny electrical charges that result from the activity of the brain cells. The charges are amplified and appear as a graph on a computer screen or as a printed recording. Sleep loss, fatigue and poor quality of sleep have been reported on numerous space missions [21]. During the space shuttle era, astronauts usually had between 5–6 h of sleep and lesser in the case of emergencies [22]. On long-duration missions, there can be changes in the quality of sleep. Problems related to this may appear and compromise the performance levels of astronauts [23,24]. Although the use of drugs is not indicated in general in the aviation work environment, some sleep medication has been used in long-duration missions upon the approval of the medical team. These sleep problems seem to be related to the lack of environmental cues, such as natural light, which produces circadian rhythm disturbances and consequent psycho-physiological effects. However, other factors, not directly related to the environmental aspects of space missions, may play a role in sleep problems, including anxiety, workload, stress or isolation. Several sleep studies using EEG tests during Columbia and NeuroLab missions showed contradictory results [25]. Sleep patterns were not substantially altered in space compared to prior mission tests; but, a reduction of total sleep was registered, and a clear alteration of circadian cycle was observed [22].

Cheron *et al.* [26] examined the alterations of alpha cortical activity during the experience of weightlessness in space and showed an increase of power in the peak alpha frequency (PAF) activity. PAF is the most dominant rhythm in the relaxed, eyes-closed state and is regarded as a marker of cortical activity. Furthermore, this oscillation is considered to be involved in mental and cognitive processes [27,28]. As there is an inverse relationship between PAF power amplitude and the blood oxygen level-dependent signal [29], it is hypothesized that the alpha power increase during the stay at the ISS is due to a general lowering of cerebral blood oxygenation of astronauts and cosmonauts undergoing weightlessness, as recently shown by Schneider *et al.* [30]. Although it has been argued that impairments in cognitive and perceptual motor performance in weightlessness are caused by changes in cerebral blood flow leading to changed electro-cortical signals registered on EEG, there currently is no evidence that a systemic shift of blood volume to the brain during weightlessness is correlated with neural activity [1,30,31].

**Figure 3:**
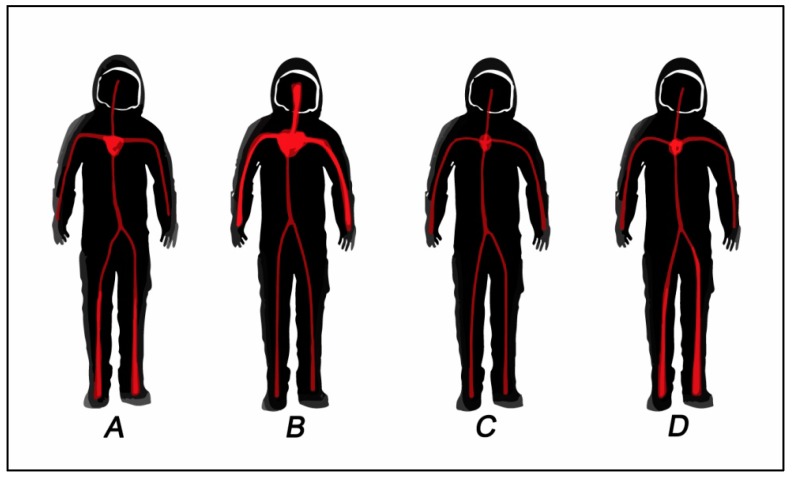
The effect of space on blood circulation. (**A**) Normal gravity (Earth); (**B**) acute zero-microgravity exposure (first day in space); (**C**) prolonged zero-microgravity exposure; (**D**) upon return to Earth.

Consequences of chronic bed-rest depend on the duration and the level of inactivity. As in weightlessness, the circulation is rearranged during the prolonged maintenance of a supine position. Initially, the central blood volume increases; perfusion and hydrostatic pressure in the lower half of the body decreases, and the slightly higher preload and stroke volume can lead to bradycardia, increased renal blood flow and mild polyuria. Over the course of weeks and months, the plasma volume and the efficacy of orthostatic reflexes regulating blood pressure decrease. When the astronaut is back on Earth again, the low blood volume is insufficient to maintain cerebral blood flow in an orthostatic position. Therefore, orthostatic hypotension may develop and dizziness may appear. A comprehensive return accommodation is required, and astronauts should be monitored for some time after prolonged time in space (Figure 3).

Nevertheless, it so far remains unclear whether neurocognitive or neuropsychological impairments and changes are provoked by microgravity itself or by secondary, environmental-related factors. In the first study using low-resolution brain electromagnetic tomography (LORETA) in low gravity [32], it was demonstrated that the microgravity phases during parabolic flights result in considerable changes in frontal lobe activity, a brain region that is known to play a major role in emotional processing and the modulation of performance [33,34].

#### 2.2.2. Space Neuropsychology

Neuropsychology is a branch of psychology that studies human behavior as it relates to normal and abnormal functioning of the CNS. Within neuropsychology, this functioning of the CNS is commonly divided into different areas, or also called higher functions, such as attention, memory, executive, language, *etc*. Unfortunately, there has not been much research in this field within space research to this date, although some data do exist. These functions can be affected in space and lead to a decrease in performance. More permanent or serious neuropsychological deficits may signal some type of brain injury. For this reason, a neuropsychological assessment and monitoring of crew neuropsychological performance is very important for mission success and crew health.

Among all neuropsychological aspects, attention is one of the most important. Attention is a complex cognitive function that is essential for human behavior. Attention is a selection-based process required to maintain an external (sound, image, smell, *etc.*) or internal (thought) event at a certain level of awareness. It is not a stable, but, rather, a fluctuating skill. It is not continuously sustained, often subconsciously let up during a task. For a further review, studies on attention were performed during the Soyuz/Salyut (26/6 and T5/7) missions in the 1970s and 1980s [18].

Other neurocognitive aspects commonly affected in space are spatial orientation, mental rotation and recognition, spatial perception and representation and other perceptual skills (Table 2). Most perceptual problems are related to the microgravity environment characteristics that make astronauts see objects in non-customary orientations. In addition, the interaction of spatial perception with the vestibular system can be a source of conflicts in neural processing, as explained previously. Proper perception of objects may be negatively affected by non-customary orientations. One well-known example is the perception of faces. This problem also exists in space, and it is easy to understand how this may have an effect on face-to-face communication. However, other aspects of perception, such as perceived verticality, as mentioned before, are not affected. This effect is defined as the difficulty of face recognition when a face is inverted [19,35].

More recently, research has focused on developing assessment tools to detect and monitor these deficits and problems and counteract them. A decline in attentiveness may primarily occur in space, because of stress-related factors. Problems in attention performance can also indicate the possible compromise of other neuropsychological aspects, such as memory. Having accurate and helpful assessment tools is very important for monitoring performance levels and the mental health of astronauts in space. The unique environmental constraints and characteristics of space have required the development of some specific tools over the years to assess and combat these issues. Moreover, this continues to be an important field of research. Although some now dated cognitive tests and batteries, such as MINICOG [36] or the AGARD [37] test, have been used in space and simulations before. The Spaceflight Cognitive Assessment Tool for Windows (WinSCAT) is the current standard for this type of assessment in space operations. WinSCAT is a time-constrained test of cognitive abilities, such as attention, math and memory [38]. Right now, WinSCAT is routinely performed by astronauts aboard the ISS every 30 days, before or after their periodic health status test. It is also administered on special crewmembers upon the flight surgeons’ request.

**Table 2:** Summary of brain areas, functions associated with and symptoms related to weightless/space conditions.

Brain Area (*)	Function	Symptom
Primary somatosensory cortex (1, 2, 3)	Proprioception Somatic sensations	Somatosensory problems, self-position accuracy problems
Parietal cortex, somatosensory association cortex (5, 7)		
Primary and association visual cortex (17, 18)	Visual perception	Color perception problems, loss of acuity
Auditory association cortex (22)	Hearing and auditory perception	Sound localization in binaural hearing
Prefrontal cortex and premotor cortex (11, 47, 6)	Problem solving, executive functions, working memory, task management, inhibitory control, decision making and attention	Executive problems: decision-making errors, attention problems, spatial working memory, concentration problems
Primary motor cortex (4)	Voluntary motor initiation, especially in the distal extremities and facial and oral musculature	Difficulty acquiring targets in voluntary movements, transient effects during first month
Frontal eye field (8)	Non-tracking voluntary eye movements, visual attention	Visual attention problems
Cerebellum	Motor control	Motor coordination and movement-timing problems
Entorhinal cortex, olfactory cortex, insula (34)	Olfaction, taste and memory	Perceived changes in taste and smell of food
Vestibular system and cortex	Gravity-sensing, 3D positioning in space, and sensory orientation-integration.	Space motion sickness, malaise, headache, vomiting, lack of motivation and dizziness
Limbic system	Emotions, social behavior, attention, memory, motivation, olfaction, learning, decision-making and reward sensitiveness	Diminished social interaction, irritability, concentration problems, lack of motivation, memory problems, depression, anxiety and mood problems
Brainstem	Sleep cycle and arousal	Sleep problems

* Numbers indicate Brodmann’s area codes (see Figure 1).

### 2.3. Psychosocial and Neurobehavioral Aspects

Astronauts must maintain a high level of performance efficiency over the course of their stay in space. During space missions, astronauts are exposed to an environment that can induce detrimental effects on mood and performance. This is confirmed by a number of studies that report impacts on mood, performance, workload and social aspects of long-term habitation in space [9,39,40,41,42]. There are thus many reasons to perform neurocognitive assessments in space. The crew has to be prepared in case of any possible events or conditions that may affect neurocognitive performance and subsequently compromise mission success or survival. The unique environmental characteristics of space missions may affect performance, and some of these characteristics are present in simulations. By way of example, during the Mars-500 experiment, subjects experienced isolation, limited space, communication delays with mission control, *etc*. Other challenges of great importance that are commonly faced during habitation in space cannot be simulated (e.g., radiation and the impossibility of rescue). Neurocognitive and neurobehavioral problems occurring in space can be mainly related to four different sources, according to Kanas and Manzey [19]: (1) physical factors, including acceleration, microgravity, radiation and light/dark cycles; (2) habitability factors, including vibration, noise, temperature, light and air quality; (3) psychological factors, including isolation, danger, monotony and workload; (4) social or interpersonal factors, including gender issues, cultural effects, crew size, leadership issues and personality conflicts.

Cognitive performance should not be separated from broader human social nature. Social aspects play a key role in space missions. Impaired physical and social interactions may result in problems for the crew, especially in long-duration missions. These issues may result in several potentially hazardous conditions, such as a loss of motivation, loneliness, lower performance, depression or other medical conditions [43]. Anecdotal information available from Russian missions reveals that these problems do appear in space missions and are not always very clearly recognized or understood by crewmembers. These aspects have also been studied in analogous situations on Earth [44,45,46].

Regarding psychiatric problems, some evidence is also available from both space missions and analog missions on Earth, such as Antarctic stations and submarine research stations. In these analogous scenarios and simulations, not frequently, but sometimes, we were able to observe how chronic isolation may lead to depression, negative adjustment reactions and psychosomatic problems in members of small teams [19,47]. In addition, other conditions, like frustration, perceived inability to change things and low light levels, may lead to depression.

The limbic system plays a key role in the emotional responses of mammals and humans. These areas together with frontal cortex and other relevant areas of the brain regulate socio-emotional life and mood. Changes in mood are normal and sometimes may be hard to detect before they evolve into a clinical condition. In small work groups, it may be difficult to perceive these changes in time, and they are usually detected after they already interfere with performance. It is worth noting here that depression may even terminate a space mission, as was the case with Salyut 7 in 1985. Anxiety is also a common problem on Earth, and it has been detected in astronauts on Antarctic missions. It is, however, not common in space missions, though it may appear, as indicated by some Mir missions [19,48]. The problem with anxiety is its delayed detection. This is why tools for monitoring and treating early symptoms should be researched in greater depth.

The condition known as asthenia is an important issue in mental health in space [19]. It is a controversial topic though, because there is no agreement between space agencies. For Russians, asthenia is a real syndrome, and it is used to describe a set of psychological changes that commonly occur among astronauts. The principle symptoms are fatigue, dizziness, tension headaches, sleep disturbances and/or irritability. In contrast, this diagnosis is not presently recognized in the American DSM [49]. The core feature of all of these syndromes or symptoms is that they may somehow affect the performance of the crew, and we know that lower performance levels may compromise mission success. It is therefore essential that space research pays attention to how these emotional, stress-associated and psychological issues affect cognitive performance, particularly regarding control processes, such as the perception of time [50,51], the relationship between automatic and controlled processes (inhibitory processes) and the categorization of incoming information [52,53].

A final and very interesting factor is the relationship between ground control and crew, about which little is known in long-duration space missions. Some Mir, Skylab and Apollo data [54] and simulations [55] show that this can be a significant source of problems. There is evidence that during some Mir and ISS missions, on-board crew tension was outwardly displayed to mission control, which may have negatively affected the crew-ground relationship [56]. In long-duration missions, ground control risks losing an accurate sense of the situation in the spaceship. This is especially concerning when considering the delays in communications that will exist, for example, in a mission to Mars, which may go above 37 min. Although some pilot research exists on the differences between high *versus* low autonomy environments in simulations and analogs [46], further research is needed on aspects of astronaut autonomy, especially as it relates to mental health maintenance and monitoring, for future long-duration missions, such as travel to Mars. Finally, we would like to mention the positive effects on behavioral health that spaceflight may also have on crewmembers. The overview effect consists of a cognitive shift in awareness reported by some astronauts and cosmonauts during spaceflight [57]. It refers to the experience of seeing Earth from space and the sudden awareness of the planet conception with no boundaries and the need to create a planetary society to protect it. This is proof that even stressful space environment also produce positive effects on a human’s mind. These positive effects are related to the term of salutogenesis. Salutogenesis refers to processes by which powerful experiences enhance or bring about well-being and personal growth [19]. Isolated and extreme environments can produce these salutogenesis effects, for example, in polar stations [58,59] and in space simulations [60].

## 3. Conclusions

Back in 1961, Soviet scientists were worried that a prolonged period of weightless could be fatal. This is why they decided to limit pioneer Yuri Gagarin’s first space flight to just 108 min and a single orbit. Now, we know that the human body behaves oddly in orbit, because of the lack of gravity, among other factors. However, after decades of space flight, we now understand that humans can adapt to life in this completely new environment for moderately long periods. Aside from the sporadic human visits to the Moon by the Apollo missions, ISS may represent the more continuous and representative example of the human presence in space. Surely, new steps are to come, culminating in interplanetary space missions and the establishment of human bases on other planets or moons in our solar system. Indeed, the survival of humanity may depend on this. However, major challenges await, and more research has to be done to further understand the effects that the completely new environment of space and the celestial bodies of our solar system will have on human biology and the mind. We are getting a clearer understanding of these effects on the physical body, but much more needs to be done in regard to neurocognitive and psychological phenomena, especially in the context of long-duration missions. Cognition represents a very important domain of these neural processes. Knowing how our brain and mind adapt to the space environment is crucial to understanding all factors involved in human space flight, including those related to cognition, psychology and neuroscience. In recent years, we have seen a surge in space tourism, making the space travel experience available to a greater number of people, thus allowing experts more opportunities to study these effects. Moreover, the aforementioned analogs of space, such as, Antarctic stations and desert outposts, represent good approximations of space missions, especially for the study of the psychosocial effects. The conditions faced by the crew working in these physically challenging, yet scientifically stimulating, environments are similar to those faced by astronauts in small spacecraft working in or beyond Earth’s orbit during a real space mission.
